# Non-typeable *Haemophilus Influenzae* detection in the lower airways of patients with lung cancer and chronic obstructive pulmonary disease

**DOI:** 10.1186/s40248-018-0123-x

**Published:** 2018-04-09

**Authors:** Krishna B. Sriram, Amanda J. Cox, Pathmanathan Sivakumaran, Maninder Singh, Annabelle M. Watts, Nicholas P. West, Allan W. Cripps

**Affiliations:** 1 Department of Respiratory Medicine, Gold Coast University Hospital, 1 Hospital Boulevard, Southport, QLD 4215 Australia; 2 School of Medicine, Griffith University, Southport, Australia; 3 Menzies Health Institute Queensland, Griffith University, Southport, Australia; 4 School of Medical Science, Griffith University, Southport, Australia

**Keywords:** Non-typeable Haemophilus Influenzae, Lung Cancer, COPD

## Abstract

**Background:**

Chronic airway inflammation and hypersensitivity to bacterial infection may contribute to lung cancer pathogenesis. Previous studies have demonstrated that nontypeable *Haemophilus influenzae* (NTHi) is the most common colonizing bacteria in the lower airways of patients with COPD. The objective of this study was to determine the presence of NTHi and immunoglobulin concentrations in patients with lung cancer, COPD and controls.

**Methods:**

Serum and bronchial wash samples were collected from patients undergoing diagnostic bronchoscopy. Total IgE, IgG and specific NTHi IgG were measured by enzyme linked immunosorbent assay. Bronchial wash samples were examined for the presence of NTHi via PCR.

**Results:**

Out of the 60 patients: 20 had confirmed Lung Cancer, 27 had COPD only and 13 were used as Controls. NTHi was detected in the lower airways of all three groups (Lung Cancer 20%; COPD 22% and Controls 15%). Total IgE was highest in Lung Cancer subjects followed by COPD and control subjects (mean ± SD: 870 ± 944, 381 ± 442, 159 ± 115). Likewise total IgG was higher in Lung cancer (Mean ± SD: 6.99 ± 1.8) patients compared to COPD (Mean ± SD: 5.43 ± 2).

**Conclusions:**

The lack of difference in NTHi and specific antibodies between the three groups makes it less likely that NTHi has an important pathogenetic role in subjects with Lung Cancer. However the detection of higher IgE antibody in Lung Cancer subjects identifies a possible mechanism for carcinogenesis in these subjects and warrants further study.

**Electronic supplementary material:**

The online version of this article (10.1186/s40248-018-0123-x) contains supplementary material, which is available to authorized users.

## Introduction

Lung cancer is the leading cause of cancer related mortality, accounting for over one million deaths worldwide annually [1]. Chronic Obstructive Pulmonary Disease (COPD) is the fourth leading cause of death worldwide, with an estimated prevalence of 4–10% [2]. COPD is characterised by chronic inflammation of the lower airways and similar to 90% of lung cancer cases is caused by cigarette smoking [2]. The presence of COPD can increase the risk of lung cancer by 4.5-fold [3] and among smokers with COPD, inflammation persists and lung function continues to deteriorate even following smoking cessation [4].

Some animal models have suggested that nontypeable (i.e. uncapsulated) *Haemophilus influenzae* (NTHi) may play a causal role for a COPD-like airway inflammation and in lung cancer promotion [5, 6]. Exposure of genetically modified mice to NTHi and the most potent cigarette smoke carcinogen (NNK), resulted in a 2.2-fold increase in the number of tumours identified [5]. NTHi presence may induce inflammation which may subsequently promote lung carcinogenesis. NTHi can be found in the lower respiratory tract of 30% - 50% of COPD patients [7]. To the best of our knowledge, there have not been any studies specifically evaluating the prevalence of NTHi in lung cancer patients compared with COPD patients without lung cancer. The aims of this study are to measure and compare the presence of NTHi in the bronchial airway and NTHi specific antibodies in the blood of lung cancer and COPD patients.

## Methods

### Study sample

Consecutive adult (age ≥ 18 years old) outpatients were considered for the study. All participating subjects provided written informed consent. The study was approved by the local Human Research Ethics Committee (HREC/14/QPCH/75; MSC/02/14/HREC). Power calculations were performed to determine the minimum sample size of patients with lung cancer, COPD and controls with an alpha value of 0.05 and power of 80%. Based on the reported incidence of NTHi in bronchoscopy [8] and sputum [9], the minimum sample size to detect NTHi among the groups (i.e primary outcome at an estimated clinically meaningful effect) at a power of 0.05 and 80% was 11 patients with lung cancer, 11 patients with COPD and 7 control patients. Power calculations determined that a sample size of 60 patients would provide a power coefficient of 0.96.

Consecutive patients who were required to undergo a bronchoscopy procedure were considered for the study. Patients were excluded if they were below 18 years old, unable to provide written consent, if the bronchoscopy procedure was being performed as a medical emergency, or if the procedure was performed on the weekend or outside usual working hours (8 am-5 pm). All patients scheduled to undergo bronchoscopy were given standardized written and verbal information about the research study. Patients who were experiencing a lower respiratory tract infection or COPD exacerbation were excluded from the bronchoscopy procedure and hence the study as well. Consenting patients were recruited to the study. Contact was made with eligible participants between November 2014 and September 2015.

### Biological samples

All three groups of patients had bronchial wash and serum samples collected. The bronchial wash samples consisted of two aliquots of at least 10 mls of sterile normal saline obtained from the right middle lobe. One aliquot of the bronchial wash was sent for routine bacterial culture at the Clinical Microbiology Department, Queensland Pathology, Gold Coast University Hospital. The second aliquot was sent for NTHi polymerase chain reaction (PCR) analysis. Briefly, DNA was extracted from bronchial wash samples using a salt and alcohol precipitation method [10]. A unique primer and probe set was used to detect the fucP locus of the NTHi genome as described previously [11]. A second reaction for the detection of the *bexA* locus, characteristic of typeable haemophilus strains, was also performed as described previously [12]. Samples were classified as NTHi positive, based on the detection of the *fucP* locus in the absence of the *bexA* locus.

Blood was collected by venepuncture immediately before bronchoscopy. The blood was clotted and the serum separated by centrifugation. Total IgE concentrations were determined using commercially available enzyme linked immunosorbent assay (ELISA) kits according to the manufacturer’s instructions (Affymetrix eBioscience, San Diego, CA, USA). NTHi-specific IgG antibodies to both total NTHi lysate and an outer membrane protein (OMP) preparation were measured in the serum using commercially available enzyme linked immunosorbent assay reagents (ELISA; Affymetrix eBioscience, San Diego, CA, USA). Briefly, 96-well Nunc Maxisorp immunoassay plates (Affymetrix eBioscience, San Diego, CA, USA) were coated overnight with 100 ng of either complete lysate or OMP, in a carbonate buffer (15 mM Na2CO3, 35 mM NaHCO3) prepared as described previously [13]. Assays were then completed using standardised protocols as per manufacturer’s instructions (Affymetrix eBioscience, San Diego, CA, USA)..

All statistical analyses were performed using SPSS (Version 19, SPSS Inc., Chicago, USA). Kurtosis and skewness evaluation was performed to determine if measurements had normal distribution. Normally distributed variables are reported as means and standard deviations. Comparative analysis of normally distributed data and binomial data was performed using one-way ANOVA and Chi-Square test, respectively. Statistical significance was accepted at *p* < 0.05.

## Results

### Subjects

After exclusions, sixty subjects provided written consent and were recruited for the study. In the study cohort, 20 patients had been diagnosed with lung cancer (confirmed pathological diagnosis of lung cancer), 27 with COPD (Spirometry confirmed diagnosis of COPD AND chest x-ray or CT within the preceding 3 months to exclude lung cancer diagnosis) and 13 subjects were the control group (Spirometry and imaging excluded diagnosis of lung cancer and/or COPD). Characteristics of the groups are shown in Table 1. The three groups were matched for age and gender. The prevalence of never smokers was highest in the Control patients (69%) and lowest in the Lung Cancer patients (5%). Lung function measurements demonstrated that the FEV_1_ (mean age ± SD) was significantly different between the three groups of patients (Lung Cancer patients: 67 ± 17; COPD patients: 68 ± 25; Control patients: 91 ± 20, *p* = 0.007). There was no difference in the frequency of diagnosis of asthma across the three groups of patients (*p* = 0.385). With regards to respiratory medications, there were no differences across the groups for inhaled corticosteroids (*p* = 0.290), inhaled bronchodilators (*p* = 0.241) and systemic corticosteroids (*p* = 0.228). No patients were receiving systemic antibiotics at the time of the study.

**Table 1:** Characteristics of Study Subjects

Characteristics	Lung Cancer	COPD	Control
Number of subjects	20	27	13
Age (mean ± SD)	67 ± 10	68 ± 9	58 ± 15
Males, n (%)	10 (50)	17 (63)	6 (46)
Asthma diagnosis, n (%)	3 (15)	5 (19)	3 (23)
Spirometry (mean ± SD)			
FEV_1_ (% of predicted)	67 ± 17	68 ± 25	91 ± 20
FVC (% of predicted)	86 ± 12	88 ± 24	91 ± 20
FEV_1_/FVC	59 ± 12	56 ± 12	78 ± 6
Smoking status, n(%)			
Current smoker	13 (65)	7 (26)	0
Former smoker	6 (30)	18 (67)	4 (31)
Never smoker	1 (5)	2 (7)	9 (69)
Pack/years in smokers (mean ± SD)	44 ± 18	43 ± 28	8 ± 15
Treatment at time of review, n (%)			
Antimicrobial therapy	0	0	0
Inhaled corticosteroid therapy	5 (25)	11 (41)	4 (31)
Inhaled bronchodilator therapy	6 (30)	13 (48)	4 (31)
Systemic corticosteroid therapy	1 (5)	0	1 (7)

*Abbreviations: FEV*_*1*_ Forced Expiratory Volume in one second, *FVC* Forced Vital Capacity, *NTHi* Non-Typeable Haemophilus

### NTHi identification

Using the PCR method, NTHi was identified in the bronchial wash samples of 20% of Lung Cancer patients, 22% of COPD patients and 15% of Control patients (Table 2). Conversely, using the culture method, NTHi was only identified the bronchial wash samples of 10% of Lung Cancer patients, 22% of COPD patients and 8% of Control patients (*p* = 0.36). There was a strong correlation between the NTHi PCR and culture results (r^2^ = 0.56, *p* < 0.001).

**Table 2:** Biomarkers in the Lung cancer, COPD and Control Groups

Biomarker	Lung Cancer	COPD	Control
	*N* = 20	*N* = 27	*N* = 13
Serum,			
Total IgG ng/ml (mean ± SE)	6.87 ± 0.38**	5.36 ± 0.39	5.97 ± 0.51
Total IgE, ng/ml (median ± IQR)	243 ± (181.3–305.5)†, **	172.5 ± (95.5–297.8)*	91.0 ± (69.5–266.5)
IgG to NTHi OMP, RU (mean ± SE)	2.10 ± 0.22	2.02 ± 0.16	1.74 ± 0.24
IgG to NTHi P6, (mean ± SE)	1.55 ± 0.13	1.62 ± 0.10	1.32 ± 0.07
Bronchial wash (n (%)			
NTHi Culture positive	2 (10)	6 (22)	1 (8)
NTHi PCR positive	5 (20)	6 (22)	2 (15)
PPM			
Staphylococcus aureus		1	
Streptococcus pneumoniae		1	
Pseudomonas aeruginosa		2	
Stenotrophomonas maltophilia		1	
Non-PPM			
Atypical Mycobacterium			1
Aspergillus fumigatus	1	1	
No microorganism	17	16	11

*p < 0.05 between COPD and controls; † *p* < 0.05 between Lung Cancer and controls; ** *p* < 0.05 between Lung Cancer and COPD

PPM-Potential Pathogenic Organisms

### Serum immunoglobulins

The immunoglobulin differences between the three groups in the study are provided in Table 2. In patients with Lung Cancer, the levels of total IgG in serum were elevated compared to COPD patients (*p* = 0.02). The total IgE levels in Lung Cancer patients were higher than COPD patients (*p* = 0.03) and control patients (91 (69.5–266.5, *p* = 0.001) respectively. Furthermore, the only difference between COPD patients and Control patients was the total IgE (*p* = 0.001). There were no other significant differences in the serum biomarkers among the three groups of patients.

## Discussion

In this study, we prospectively investigated the lower airways for NTHi bacteria in lung cancer, COPD and control patient groups. We also evaluated immunological biomarkers in the blood of the three patient populations. While there was no difference in the rates of identification of NTHi among the three patient populations, there were significant differences in immunological measures between groups.

Recently there has been growing awareness of the importance of NTHi in the pathophysiology of chronic lower respiratory tract inflammatory disorders, particularly COPD [14]. The use of bronchial wash specimens and molecular identification techniques allowed for assessment of NTHi directly from the lower airways in the current study. Bronchial colonisation by bacteria in patients with lung cancer has previously been reported [15]. Laroumagne et al. reported that bacterial bronchial colonisation can be identified in 48% of patients with lung cancer and specifically, NTHi was identified in 4.3% [8]. In the current study NTHi was detected in up to 22% of collected samples. This higher rate of identification of NTHi than that previously reported may be due to use of more sensitive methods employed to detect NTHi. However, the small sample size of our study limits our ability to make any definitive inferences about NTHi bacteria identification and possible pathogenic role of this bacteria in patients with lung cancer compared to other patient groups. A pertinent challenge will be the disentanglement of COPD from lung cancer in order to determine the pathophysiological role of NTHi. Recent evidence suggests that lung cancer patients with COPD may have a different pattern of driver mutations and molecular features [16]. It is hypothesised that these differences may be due to COPD specific inflammatory microenvironment and may result in epigenetic and other molecular mechanisms eventually promoting carcinogenesis [16]. Future studies will need to determine if NTHi colonisation contributes to the enhancement of the COPD related inflammation in the airways and lung parenchyma.

Immunoglobulin assessments revealed significantly higher total IgE and IgG concentrations in lung cancer patients, however the relevance of these observations is not clear. A possible association between allergy and cancer has been reported previously [17, 18], but widely replicated results are lacking with some studies reporting a negative association [17, 18], others showing a positive association [19, 20] or no general association [21]. Studies that have specifically evaluated IgE in patients with lung cancer have found a positive association with not only lung cancer diagnosis [22] but also increased mortality in females [23]. A possible explanation for this phenomenon is thought to be that the lung is directly exposed to the noxious stimuli which can be both allergens and carcinogens. This direct exposure induces excessive inflammation in allergic subjects which in turn may promote tumour development [24]. It is also possible that the elevated IgE levels may be an immunological response to infection. Indeed, there is now emerging data to suggest that the total microbial load is higher and considerably varied in lung cancer patients compared to controls [25]. However, it is also possible that IgE is an epi-phenomenon in patients with underlying reactive airways disease, i.e. asthma. While we obtained information about a diagnosis of asthma, we did not systematically exclude other causes of elevated IgE. Furthermore, the diagnosis of asthma was a clinical diagnosis rather than subjecting every patient to a thorough clinical and pulmonary function laboratory assessment for asthma. But there are several possible causes of an elevated IgE, such as primary immunodeficiency syndromes, infections, inflammatory disorders and malignancy, and it was beyond the scope of this study to systematically exclude other possible causes of an elevated IgE. This should be explored in future studies, where the patient populations should be controlled for (at least) the common causes of an elevated IgE.

Our study was a pilot study with a relatively small sample size. Also the results of our study were not corrected for age, which may be relevant since our cohort of patients were older than the controls. It is possible that a trend for higher prevalence of NTHi in patients with lung cancer may not have been identified in a small subgroup analysis. A larger prospective study of patients may need to be undertaken to address this limitation. Our study is an observational cohort study and consequentially there was no blinding of the study subjects to the study investigators. Future research may need to be designed such that there is adequate blinding of the study investigators and be performed across multiple centres to ensure that the results obtained are not only robust but also reproducible.

## Conclusions

Our pilot study found that the NTHi identification in the lower airways of patients with Lung Cancer is not different from patients with COPD and Controls. Further research on a larger sample size and other biological samples may be required to better characterize the prevalence NTHi in the lower airways of patients with lung cancer compared to patients with COPD and healthy individuals. It is important to continue to gain more information about NTHi in patients with lung cancer since it may contribute to the growing body of knowledge about the different mechanisms that result in lung carcinogenesis.

## Additional file

Additional file 1: Raw data of the study participants. (XLS 35 kb)

## References

[1] Jemal A, Center MM, DeSantis C, Ward EM (2010). Global patterns of Cancer incidence and mortality rates and trends. Cancer Epidemiol Biomark Prev.

[2] Decramer M, Janssens W, Miravitlles M (2012). Chronic obstructive pulmonary disease. Lancet.

[3] Punturieri A, Szabo E, Croxton TL, Shapiro SD, Dubinett SM (2009). Lung Cancer and chronic obstructive pulmonary disease: needs and opportunities for integrated research. J Natl Cancer Inst.

[4] Shapiro SD (2001). End-stage chronic obstructive pulmonary disease: the cigarette is burned out but inflammation rages on. Am J Respir Crit Care Med.

[5] Barta P, Van Pelt C, Men T, Dickey B, Lotan R, Moghaddam SJ (2012). Enhancement of lung tumorigenesis in a Gprc5a knockout mouse by chronic extrinsic airway inflammation. Mol Cancer.

[6] Moghaddam SJ, Li H, Cho S-N, Dishop MK, Wistuba II, Ji L, et al. Promotion of lung carcinogenesis by chronic obstructive pulmonary disease–like airway inflammation in a K-ras–induced mouse model. Am J Respir Cell Mol Biol. 2009;40:443–53. 10.1165/rcmb.2008-0198OC PMC2660561 18927348

[7] Sethi S, Murphy TF (2001). Bacterial infection in chronic obstructive pulmonary disease in 2000: a state-of-the-art review. Clin Microbiol Rev.

[8] Laroumagne S, Lepage B, Hermant C, Plat G, Phelippeau M, Bigay-Game L, et al. Bronchial colonisation in patients with lung cancer: a prospective study. Eur Respir J. 2013;42:220–9. 10.1183/09031936.00062212 23100491

[9] DC O, RL C, AW C (2011). Haemophilus influenzae and smoking-related obstructive airways disease. Int J COPD.

[10] Willner D, Daly J, Whiley D, Grimwood K, Wainwright CE, Hugenholtz P (2012). Comparison of DNA extraction methods for microbial community profiling with an application to pediatric Bronchoalveolar lavage samples. PLoS One.

[11] Price EP, Sarovich DS, Nosworthy E, Beissbarth J, Marsh RL, Pickering J, et al. Haemophilus influenzae: using comparative genomics to accurately identify a highly recombinogenic human pathogen. BMC Genomics. 2015;16:1–10. 10.1186/s12864-015-1857-x PMC4551764 26311542

[12] Wroblewski D, Halse TA, Hayes J, Kohlerschmidt D, Musser KA (2013). Utilization of a real-time PCR approach for Haemophilus influenzae serotype determination as an alternative to the slide agglutination test. Mol Cell Probes.

[13] Kyd JM, Taylor D, Cripps AW (1994). Conservation of immune responses to proteins isolated by preparative polyacrylamide gel electrophoresis from the outer membrane of nontypeable Haemophilus influenzae. Infect Immun.

[14] King PT, Sharma R (2015). The lung immune response to Nontypeable Haemophilus influenzae (lung immunity to NTHi). J Immunol Res.

[15] Ioanas M, Angrill J, Baldo X, Arancibia F, Gonzalez J, Bauer T, et al. Bronchial bacterial colonization in patients with resectable lung carcinoma. Eur Respir J. 2002;19:326–32. 10.1183/09031936.02.00236402 11866014

[16] Balestro E, Baraldo S, Piloni D, Stella G (2016). Lung tumors, COPD and immune response: is epigenetic the bottom line?. Minerva Med.

[17] Turner MC, Chen Y, Krewski D, Ghadirian P (2006). An overview of the association between allergy and cancer. Int J Cancer.

[18] Wang H, Diepgen TL (2005). Is atopy a protective or a risk factor for cancer? A review of epidemiological studies. Allergy.

[19] Wang H, Rothenbacher D, Löw M, Stegmaier C, Brenner H, Diepgen TL (2006). Atopic diseases, immunoglobulin E and risk of cancer of the prostate, breast, lung and colorectum. Int J Cancer.

[20] Ji J, Shu X, Li X, Sundquist K, Sundquist J, Hemminki K (2009). Cancer risk in hospitalised asthma patients. Br J Cancer.

[21] Merrill RM, Isakson RT, Beck RE (2007). The association between allergies and cancer: what is currently known?. Ann Allergy Asthma Immunol.

[22] Hällgren R, Nόu E, Arrendal H, Hiesche K (1982). Smoking and circulating IgE in bronchial carcinoma. Acta Med Scand.

[23] Taghizadeh N, Vonk JM, Hospers JJ, Postma DS, de Vries EGE, Schouten JP, et al. Objective allergy markers and risk of cancer mortality and hospitalization in a large population-based cohort. Cancer Causes Control. 2015;26:99–109. 10.1007/s10552-014-0489-9 PMC4282688 25388801

[24] Mensinga TT, Schouten JP, Rijcken B, Weiss ST, Speizer FE, Lende R. The relationship of eosinophilia and positive skin test reactivity to respiratory symptom prevalence in a community-based population study. J Allergy Clin Immunol. 1990;86:99–107. 10.1016/s0091-6749(05)80129-0 2370392

[25] Hosgood HD, Sapkota AR, Rothman N, Rohan T, Hu W, Xu J, et al. The potential role of lung microbiota in lung cancer attributed to household coal burning exposures. Environ Mol Mutagen. 2014;55:643–51. 10.1002/em.21878 PMC4217127 24895247

